# Real‐Time Prediction of Correct Yoga Asanas in Healthy Individuals With Artificial Intelligence Techniques: A Systematic Review for Nursing

**DOI:** 10.1002/nop2.70278

**Published:** 2025-08-06

**Authors:** Gözde Özsezer, Gülengül Mermer

**Affiliations:** ^1^ Faculty of Health Sciences, Department of Public Health Nursing Çanakkale Onsekiz Mart University Çanakkale Turkey; ^2^ Faculty of Nursing, Department of Public Health Nursing Ege University Turkey

**Keywords:** artificial intelligence, nursing, posture, systematic review, yoga

## Abstract

**Aim:**

This study aims to systematically review the real‐time prediction of yoga asanas using artificial intelligence (AI) techniques to improve the quality of life in healthy individuals.

**Design:**

Systematic review.

**Methods:**

A comprehensive literature review was conducted in English using the keywords ‘yoga’, ‘asana’, ‘pose’, ‘posture’, ‘machine learning’, ‘deep learning’ and ‘prediction’ in the Web of Science, Google Scholar, PubMed and Scopus databases. The objective was to identify all relevant studies on the topic. Two independent researchers screened the titles and abstracts of the retrieved publications, applying the JBI Critical Appraisal Checklist for Diagnostic Test Accuracy Studies for quality assessment. The initial search yielded 3250 studies (Google Scholar: 3190, PubMed: 19, Scopus: 27, Web of Science: 14). After applying inclusion criteria, 15 studies were included in the final systematic review.

**Results:**

Among the included studies, nine employed deep learning (DL) models, three utilised machine learning (ML) and three applied a combination of both DL and ML techniques. The primary statistical evaluation method for real‐time prediction was accuracy across all studies. The highest accuracy rates were observed in studies using DL models alone (min = 92.34%, max = 99.92%), followed by studies that combined DL and ML (min = 91.49%, max = 99.58%), and those using only ML (min = 90.9%, max = 98.51%). These findings indicate that integrating DL and ML models can enhance the accuracy of real‐time yoga asana prediction.

**Patient or Public Contribution:**

The findings advocate for the implementation of DL and ML models in clinical and community settings to improve the real‐time and precise prediction of yoga asanas, a well‐established evidence‐based nursing intervention for healthy individuals.

**Abbreviations**
AI: artificial intelligence
AUC: area under the curve
CNN: convolutional neural network
DL: deep learning
KNN: K‐nearest neighbour
LSTM: long short‐term memory
ML: machine learning
NB: Naïve bayes
PRISMA: preferred reporting items for systematic reviews and meta‐analyses
RF: random forest
RNN: recurrent neural network
SVM: support vector machine

## 1 Introduction

Yoga, originating in ancient India with a history of over 5000 years, is a holistic discipline that promotes physical, mental and spiritual well‐being (Chittineni et al. 2023). The term ‘yoga’ is derived from the Sanskrit word ‘yuj’, meaning ‘to unite’, signifying its role in harmonising the body and mind. (Akarsu and Rathfisch 2018). The core components of yoga include asanas (physical postures), pranayama (breathing exercises) and dhyana (meditation), all of which collectively contribute to overall well‐being (Sinha et al. 2021).

The World Health Organisation (WHO) acknowledges yoga as an effective tool for promoting physical activity and preventing diseases (Tarek et al. 2021). Additionally, it is widely recommended for reducing anxiety, alleviating mood disorders and enhancing quality of life (Ovayolu and Ovayolu 2019). Empirical evidence supports yoga's effectiveness in stress management, sleep quality improvement, anxiety and depression reduction, and the promotion of self‐care and awareness (Maşa and Ceylan 2020). Yoga's global popularity continues to rise, primarily due to its proven benefits for both physical and mental health, including improvements in blood pressure regulation, flexibility and cognitive function (Govindaraj et al. 2016; Bhattacharyya et al. 2021; Yeun and Kim 2021). Meta‐analyses further underscore the positive impact of yoga on immune function, stress reduction and quality of life, particularly in individuals with conditions such as Parkinson's disease (Ban et al. 2021; Tong et al. 2021; Cartwright et al. 2020).

The COVID‐19 pandemic and its associated restrictions highlighted the growing necessity for accessible yoga practices. Artificial intelligence (AI)‐powered systems, utilising advanced techniques such as machine learning (ML) and deep learning (DL), facilitate real‐time feedback on yoga poses, ensuring precise execution and minimising the risk of injury (Chittineni et al. 2023; Yadav et al. 2019; Kumar and Sinha 2020). These technological advancements have revolutionised yoga practice, enabling individuals to perform asanas with precision, even without in‐person instruction (Rajendran and Sethuraman 2023). AI‐powered systems continuously monitor user progress, delivering personalised guidance to enhance both safety and motivation (Kishore et al. 2022). In this context, this study aims to systematically investigate AI‐based real‐time yoga asana prediction techniques to enhance the quality of life in healthy individuals.

The research questions are as follows:

1. What AI techniques are used to predict correct yoga asanas?

How accurate is the real‐time prediction of correct yoga asanas in healthy individuals using AI techniques?

## 2 Method

This systematic review adhered to the Joanna Briggs Institute (JBI) Critical Appraisal Checklist for Diagnostic Test Accuracy Studies (Campbell et al. 2020) and the Preferred Reporting Items for Systematic Reviews and Meta‐Analyses (PRISMA) 2020 guidelines (Page et al. 2021). The inclusion criteria encompassed studies that utilised AI techniques for real‐time prediction of yoga asanas in healthy individuals. Studies that did not focus on real‐time yoga asana predictions or included participants with pre‐existing medical conditions were excluded. Two independent reviewers extracted data, with any disagreements resolved through consultation with a third reviewer. Key variables, including AI models, evaluation metrics and sample sizes, were systematically analysed. As this study is a systematic review, ethical approval was not required. The study adhered to the JBI Critical Appraisal Checklist for Diagnostic Test Accuracy Studies and PRISMA guidelines. As it did not involve human participants, ethical approval was not required.

### 2.1 Study Design

This systematic review was conducted and reported following the PRISMA 2020 guidelines (Page et al. 2021). The study protocol was registered in PROSPERO (registration number: CRD42024496824).

### 2.2 Eligibility Criteria

The inclusion and exclusion criteria were determined based on the PICOS framework. The PICOS framework includes Population (P), Intervention (I), Comparison (C), Outcomes (O) and Study design (S) (Methley et al. 2014).

Inclusion criteria for this study:

1. ***Population:*** Healthy individuals.
2. ***Intervention:*** Exercises involving the intervention of yoga asanas.
3. ***Comparison:*** Studies using ML and DL methods.
4. ***Outcomes:*** Asana prediction with ML and DL methods.
5. ***Study design:*** Original studies published in English between 2015 and 2025 that applied ML or DL methods in AI‐based yoga asana prediction were included.

Exclusion criteria for this study:

1. ***Population:*** Patients, pregnant women and children.
2. ***Intervention:*** Studies without intervention in yoga asanas.
3. ***Comparison:*** Studies not using ML and DL methods.
4. ***Outcomes:*** Studies that do not include ML and DL methods in asana prediction.
5. ***Study design:*** Studies that were not published in journals outside the specified time period, not published in journals, published in a language other than English, and for which the research type was not appropriate were excluded from the scope of the study (Supporting Information S1).

### 2.3 Information Sources and Search Strategy

A comprehensive literature search was conducted in Web of Science, Google Scholar, PubMed and Scopus between February 15 and 25, 2025. The search strategy was developed following PRISMA 2020 guidelines and included Boolean operators to refine the results. The literature search employed various combinations of the keywords ‘yoga’, ‘asana’, ‘pose’, ‘posture’, ‘machine learning’, ‘deep learning’ and ‘prediction’ across Web of Science, Google Scholar, PubMed and Scopus. In the search of databases, it was aimed at reaching all studies on the subject. Reference lists of included studies and previous systematic reviews were checked for additional research.

### 2.4 Selection of Studies

Two independent reviewers screened the titles, abstracts and full texts of the retrieved studies. Any discrepancies were resolved through discussion or consultation with a third reviewer. Studies from the literature on real‐time prediction of correct yoga asanas with ML and DL techniques were included. To enhance the reliability of the selection process, two independent reviewers screened all titles and abstracts independently. Inter‐rater reliability was assessed using Cohen's Kappa (κ), which yielded a κ value of 0.86, indicating a strong level of agreement.

**FIGURE 1:**
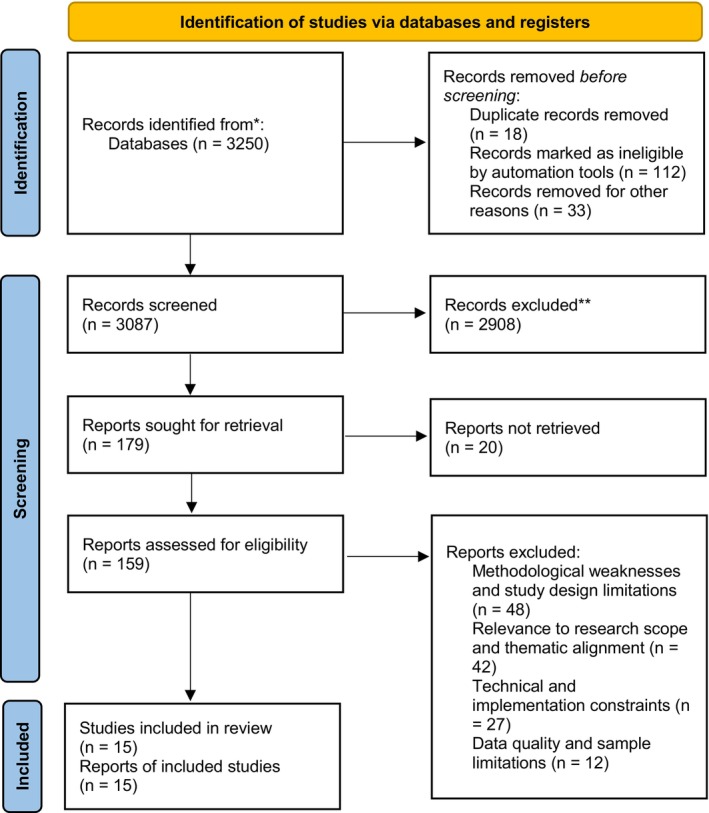
Prisma chart of search strategy and identification of article included.

The titles and abstracts of all relevant publications from the electronic searches were the subject of an independent review by researchers. The search resulted in 3250 studies (Google Scholar: 3190, Pubmed: 19, Scopus: 27, WOS: 14). The studies were first analysed according to their titles, and 2908 studies that were not related to the research topic were excluded. We excluded 148 articles that did not meet the inclusion criteria. Abstracts and full texts of the remaining 194 studies were screened for inclusion and exclusion criteria. A total of 161 studies, including reviews, letters to the editor, meta‐analyses and conference proceedings, were excluded. Duplication of 18 articles was identified and excluded. A total of 15 studies met the criteria for systematic review (Figure 1).

### 2.5 Data Extraction

To obtain the study data, a data extraction tool developed by the researchers was used. With this data extraction tool, data were extracted from the relevant studies, including the type of AI, AI model, authors, year of publication, sample size, number and type of yoga poses, statistical method used and results of the studies. The authors of the included articles were not contacted for clarification or additional data.

### 2.6 Detailed Methodological Quality Assessment of Included Studies

The methodological quality of the included studies was evaluated using the JBI Critical Appraisal Checklist for Diagnostic Test Accuracy Studies (Campbell et al. 2020). This tool evaluates 10 key domains:

1. Patient Selection—Whether a consecutive or random sample was enrolled.
2. Study Design—Avoidance of a case–control design to minimise selection bias.
3. Exclusion Criteria—Whether inappropriate exclusions were avoided.
4. Index Test Interpretation—Whether the index test results were interpreted without knowledge of the reference standard.
5. Pre‐specified Threshold—If a threshold was used, whether it was pre‐defined.

Reference Standard Validity—Whether the reference standard was appropriate to classify the target condition

6 Blinding—Whether reference standard results were interpreted without knowledge of the index test.
7 Timing Consistency—Whether there was an appropriate interval between the index test and reference standard.
8 Consistency in Reference Standard–Whether all patients received the same reference standard.
9 Inclusion in Analysis—Whether all enrolled participants were included in the final analysis.

Each study was scored from 0 to 10, with the following classification:

- High quality (≥ 7 points)
- Moderate quality (5–6 points)
- Low quality (< 5 points)

As shown in Table 1 (critical appraisal results):

- 10 out of 15 studies (67%) were classified as high quality, scoring ≥ 7.
- 3 studies (20%) were classified as moderate quality (scores of 5–6).
- 2 studies (13%) were classified as low quality, with scores below 5.

**TABLE 1:** Critical appraisal results.

Citation	Q1	Q2	Q3	Q4	Q5	Q6	Q7	Q8	Q9	Q10	Results (%)
Yadav et al. 2019	Y	Y	Y	Y	Y	Y	Y	Y	Y	Y	10/10 (100)
Kumar and Sinha 2020	Y	Y	Y	Y	Y	Y	Y	Y	Y	Y	10/10 (100)
Bahukhandi and Gupta 2021	Y	Y	Y	Y	Y	Y	Y	Y	Y	Y	10/10 (100)
Jain et al. 2021	Y	Y	Y	Y	U	Y	Y	Y	Y	Y	9/10 (90)
Anand Thoutam et al. 2022	Y	Y	Y	Y	U	Y	Y	Y	Y	Y	9/10 (90)
Bhosale et al. 2022	Y	Y	Y	Y	U	Y	Y	Y	Y	Y	9/10 (90)
Gajbhiye et al. 2022	Y	Y	Y	Y	U	Y	Y	Y	U	U	7/10 (70)
Garg et al. 2022	Y	Y	Y	Y	U	Y	Y	Y	U	U	7/10 (70)
Kishore et al. 2022	Y	Y	Y	Y	U	Y	Y	Y	Y	Y	9/10 (90)
Rathikarani et al. 2022	Y	Y	Y	Y	U	Y	Y	Y	Y	Y	9/10 (90)
Swain et al. 2022	Y	Y	Y	Y	Y	Y	Y	Y	Y	Y	10/10 (100)
Ashraf et al. 2023	Y	Y	Y	Y	U	Y	Y	Y	U	U	7/10 (70)
Palanimeera and Ponmozhi 2023	Y	Y	Y	Y	Y	Y	Y	Y	Y	Y	10/10 (100)
Srivastava et al. 2023	Y	Y	Y	Y	U	Y	Y	Y	U	U	7/10 (70)
Upadhyay et al. 2023	Y	Y	Y	Y	U	Y	Y	Y	Y	Y	9/10 (90)

*Note:* (Q1) Was a consecutive or random sample of patients enrolled? (Q2) Was a case control design avoided? (Q3) Did the study avoid inappropriate exclusions? (Q4) Were the index test results interpreted without knowledge of the results of the reference standard? (Q5) If a threshold was used, was it pre‐specified? (Q6) Is the reference standard likely to correctly classify the target condition? (Q7) Were the reference standard results interpreted without knowledge of the results of the index test? (Q8) Was there an appropriate interval between index test and reference standard? (Q9) Did all patients receive the same reference standard? (Q10) Were all patients included in the analysis? N (no) = 0; NA (not applicable) = 0; U (unclear) = 0; Y (yes) = 1.

The primary methodological weaknesses observed in lower‐quality studies included unclear sample selection criteria, lack of reference standardisation and absence of a pre‐defined threshold for AI model accuracy assessments. These limitations may introduce bias, particularly in ML and DL model performance comparisons.

### 2.7 Evidence Quality Assessment Using GRADE

To assess the certainty of evidence, we applied the Grading of Recommendations, Assessment, Development and Evaluations (GRADE) system (Guyatt et al. 2008). The GRADE approach evaluates five key domains:

- Risk of Bias—Potential systematic errors and methodological limitations in the studies.
- Inconsistency—Variability in study results across different studies.
- Indirectness—The applicability of study findings to the target population and research question.
- Imprecision—the statistical uncertainty associated with small sample sizes or wide confidence intervals.
- Publication Bias—The risk of selective reporting or underreporting of results.

**Two independent reviewers** (Author 1 and Author 2) assessed the certainty of evidence using the GRADE framework. Each study was independently rated for the five GRADE criteria. Any **discrepancies between the two reviewers** were resolved through discussion. In cases where consensus could not be reached, a **third reviewer was consulted** to finalise the rating.

## 3 Results

### 3.1 Descriptive Characteristics of the Studies

Fifteen studies met the inclusion criteria and were included in this systematic review. Figure 1 presents the PRISMA 2020 flow diagram, detailing the study selection process, including the number of records identified, screened, excluded and included. The methodological quality of the articles was high, with all scoring 70% or more. No articles were excluded based on methodological quality (Table 2).

**TABLE 2:** Characteristics of the studies included in the systematic review.

Type of AI	No	AI model	Authors	Year of publication	Sample size	Number and type of yoga poses	Statistical method used	Results
DL	1	CNN LSTM	Yadav ve ark	2019	12 individuals (7 women and 5 men)	6 yoga poses (cobra, lotus, corpse, mountain, triangle, tree)	Accuracy	The models predicted in real time with 98.92% accuracy. The approach of using CNN and LSTM is very effective
	2	CNN LSTM	Kumar & Sinha	2020	15 individuals (5 women and 10 men)	6 yoga poses (cobra, lotus, mountain, triangle, tree, corpse)	Accuracy	The deep learning model predicted with 99.92% accuracy
	3	3D‐ CNN	Jain ve ark	2021	27 individuals (19 women and 8 men)	10 yoga poses (wreath, happy baby, head and knees, crescent, standing forward bend, plank, mountain, raised arms, seated forward bend, cane)	Accuracy	The deep learning model predicted with 99.39% accuracy
	4	CNN LSTM	Swain ve ark	2022	15 individuals	6 yoga poses (cobra, lotus, corpse, mountain, triangle, tree)	Accuracy Precision Recall AUC	It was stated that the model predicted with 99.53% accuracy, the area under the AUC curve was 0.99 and the results were extremely high
	5	Transfer learning InceptionResNetV2 InceptionV3 VGG16 NASNetMobile YogaConvo2d	Garg ve ark	2022	Unspecified	5 yoga poses (downward facing dog, goddess, plank, tree, warrior I)	Accuracy Precision Recall F1 Score	VGG16 achieves the highest accuracy on non‐skeletised images (95.6%), followed by InceptionV3, NASNetMobile, YogaConvo2d (the proposed model) (89.9%) and finally InceptionResNetV2. In contrast, the YogaConvo2d model proposed using skeletonised images has an accuracy of 99.62%. It is followed by VGG16, InceptionResNetV2, NASNetMobile and InceptionV3

	6	LSTM	Palanimeera & Ponmozhi	2023	10 individuals (5 women and 5 men)	10 yoga poses (bridge, child, downward facing dog, plank, seated forward bend, mountain, tree, triangle, warrior I, warrior II)	Accuracy	Real‐time yoga pose prediction was performed with 99.02% accuracy
	7	Y_PN‐MSSD	Upadhyay ve ark	2023	5 individuals (3 women and 2 men)	7 yoga poses (cobra, chair, downward facing dog, shoulder pose, triangle, tree, warrior I)	Accuracy	The deep learning model predicted with 99.88% accuracy
	8	Transfer learning ResNet‐50 InceptionNet InceptionResNetV2 Xception YoNet	Ashraf ve ark	2023	Unspecified	5 yoga poses (downward facing dog, lotus, mountain, warrior I, warrior II)	Accuracy	In the study where the YoNet model was proposed, it was stated that the model gave higher results with 95% accuracy compared to other models
9	RNN LSTM	Srivastava ve ark	2023	Unspecified	10 yoga poses (mountain, wreath, happy baby, head and knees, plank, raised arms, cane, standing forward bend, crescent moon, seated forward bend)	Accuracy	LSTM successfully classified yoga poses with an average accuracy of 92.34
ML	10	LR SVM RF KNN NB	Bahukhandi & Gupta	2021	15 individuals	6 yoga poses (tree, cobra, mountain, lotus, triangle, corpse)	Accuracy Precision Recall F1 Score	After the data were fully preprocessed, the ML models were trained and the LR model predicted with a maximum accuracy of 94% among all models
	11	KNN	Bhosale ve ark	2022	10 individuals (5 women and 5 men)	5 yoga poses (goddess, mountain, plank, tree, warrior III)	Accuracy	The machine learning model predicted with 98.51% accuracy
	12	EpipolarPose OpenPose PoseNet MediaPipe	Kishore ve ark	2022	20 individuals	5 yoga poses (tree, triangle, half moon, mountain, warrior I)	Accuracy	It was reported that MediaPipe gave better results than the other methods and was able to predict stoppages with more accuracy (90.9%)
DL + ML	13	SVM KNN RF MLP CNN LSTM	Anand Thoutam ve ark	2022	15 individuals	6 yoga poses (tree, cobra, mountain, lotus, triangle, corpse)	Accuracy	In the present study, SVM: 93.19%; CNN: 98.58% and LSTM: 99.38% test accuracy. In the present study, MLP accuracy is much lower than CNN and LSTM, but it achieved an accuracy of 99.58% with modified features
	14	MobileNetV2 DenseNet201 SVM RF	Rathikarani ve ark	2022	10 individuals (5 women and 5 men)	5 yoga poses (downward facing dog, goddess, plank, tree, warrior II)	Accuracy	Compared to other algorithms, it was reported that the highest accuracy of 91.49% was achieved by using RF and MobileNetV2 algorithms together
	15	CNN SVM	Gajbhiye ve ark	2022	Unspecified	5 yoga poses (lotus, corpse, mountain, triangle, tree)	Accuracy	In the study, SVM: 93.19%; CNN: 98.68% test accuracy was obtained

Abbreviations: CNN, convolutional neural network; KNN, K‐nearest neighbour; LR, logistic regression; LSTM, long‐short‐term‐memory; MLP, multi‐layer perceptron; NB, Naive bayes; RF, random forest; RNN, recurrent neural network; SVM, support vector machine.

Table 2 shows that the sample was specified in 11 studies (Yadav et al. 2019; Kumar and Sinha 2020; Bahukhandi and Gupta 2021; Jain et al. 2021; Anand Thoutam et al. 2022; Bhosale et al. 2022; Kishore et al. 2022; Rathikarani et al. 2022; Swain et al. 2022; Palanimeera and Ponmozhi 2023; Upadhyay et al. 2023); four studies did not specify the sample (Garg et al. 2022; Ashraf et al. 2023; Gajbhiye et al. 2022; Srivastava et al. 2023). A total of 154 healthy individuals comprised the sample of the studies. In 7 studies, the gender of the participants was also specified, with 49 being female and 40 being male (Yadav et al. 2019; Kumar and Sinha 2020; Jain et al. 2021; Bhosale et al. 2022; Rathikarani et al. 2022; Palanimeera and Ponmozhi 2023; Upadhyay et al. 2023).

### 3.2 Yoga Asanas Used in the Studies

Twenty‐five different yoga poses were used in the included articles. Mountain (tadasana) pose in 12 studies (Yadav et al. 2019; Kumar and Sinha 2020; Bahukhandi and Gupta 2021; Jain et al. 2021; Anand Thoutam et al. 2022; Bhosale et al. 2022; Gajbhiye et al. 2022; Kishore et al. 2022; Swain et al. 2022; Ashraf et al. 2023; Palanimeera and Ponmozhi 2023; Srivastava et al. 2023), tree (vrksasana) pose (Yadav et al. 2019; Kumar and Sinha 2020; Bahukhandi and Gupta 2021; Anand Thoutam et al. 2022; Bhosale et al. 2022; Gajbhiye et al. 2022; Garg et al. 2022; Kishore et al. 2022; Rathikarani et al. 2022; Swain et al. 2022; Palanimeera and Ponmozhi 2023; Upadhyay et al. 2023), triangle (trikonasana) pose in 9 studies (Yadav et al. 2019; Kumar and Sinha 2020; Bahukhandi and Gupta 2021; Anand Thoutam et al. 2022; Gajbhiye et al. 2022; Kishore et al. 2022; Swain et al. 2022; Palanimeera and Ponmozhi 2023; Upadhyay et al. 2023), lotus (padmasana) pose in 7 studies (Yadav et al. 2019; Kumar and Sinha 2020; Bahukhandi and Gupta 2021; Anand Thoutam et al. 2022; Gajbhiye et al. 2022; Swain et al. 2022; Ashraf et al. 2023), cobra (bhujangasana) pose in 6 studies (Yadav et al. 2019; Kumar and Sinha 2020; Bahukhandi and Gupta 2021; Anand Thoutam et al. 2022; Swain et al. 2022; Upadhyay et al. 2023), corpse (savasana) pose in 6 studies (Yadav et al. 2019; Kumar and Sinha 2020; Bahukhandi and Gupta 2021; Anand Thoutam et al. 2022; Gajbhiye et al. 2022; Swain et al. 2022), plank (kumbhakasana) pose in 6 studies (Jain et al. 2021; Bhosale et al. 2022; Garg et al. 2022; Rathikarani et al. 2022; Palanimeera and Ponmozhi 2023; Srivastava et al. 2023), downward‐facing dog (adho mukha svanasana) pose in 5 studies (Garg et al. 2022; Rathikarani et al. 2022; Ashraf et al. 2023; Palanimeera and Ponmozhi 2023; Upadhyay et al. 2023), warrior I (virabhadrasana I) poses in 5 studies (Garg et al. 2022; Kishore et al. 2022; Ashraf et al. 2023; Palanimeera and Ponmozhi 2023; Upadhyay et al. 2023), Goddess (utkata konasana) pose in 3 studies (Bhosale et al. 2022; Garg et al. 2022; Rathikarani et al. 2022), sitting forward bending (paschimottanasana) pose in 3 studies (Jain et al. 2021; Palanimeera and Ponmozhi 2023; Srivastava et al. 2023), warrior II (virabhadrasana II) pose in 3 studies (Rathikarani et al. 2022; Ashraf et al. 2023; Palanimeera and Ponmozhi 2023); garland (malasana) pose in 2 studies (Jain et al. 2021; Srivastava et al. 2023); happy baby (ananda balasana) pose in 2 studies (Jain et al. 2021; Srivastava et al. 2023); head‐to‐knee bending (janu sirsasana) pose in 2 studies (Jain et al. 2021; Srivastava et al. 2023), crescent (anjaneyasana) pose in 2 studies (Jain et al. 2021; Srivastava et al. 2023), standing forward bending (uttanasana) pose in 2 studies (Jain et al. 2021; Srivastava et al. 2023), raised arms (hasta uttanasana) pose in 2 studies (Jain et al. 2021; Srivastava et al. 2023), cane (dandasana) pose in 2 studies (Jain et al. 2021; Srivastava et al. 2023), bridge (urdhva dhanurasana) pose in one study (Palanimeera and Ponmozhi 2023) and child (utthita balasana) pose in one study (Palanimeera and Ponmozhi 2023), chair (utkatasana) pose in one study (Upadhyay et al. 2023), shoulder (sarvangasana) pose in one study (Upadhyay et al. 2023), half‐moon (ardha chandrasana) pose in one study (Kishore et al. 2022) and in one study, warrior III (virabhadrasana III) pose (Bhosale et al. 2022) was found to be used (Table 2).

### 3.3 AI Models and Algorithms Used in the Study

Among the included studies, 9 employed only deep learning (DL) models (Yadav et al. 2019; Kumar and Sinha 2020; Jain et al. 2021; Swain et al. 2022; Garg et al. 2022; Ashraf et al. 2023; Palanimeera and Ponmozhi 2023; Srivastava et al. 2023; Upadhyay et al. 2023), 3 used only machine learning (ML) models (Bahukhandi and Gupta 2021; Bhosale et al. 2022; Kishore et al. 2022) and 3 incorporated both DL and ML models (Anand Thoutam et al. 2022; Gajbhiye et al. 2022; Rathikarani et al. 2022) for real‐time yoga asana prediction. In three of the studies using DL models, Convolutional Neural Network (CNN) and Long Short Term Memory (LSTM) algorithms were used together (Yadav et al. 2019; Kumar and Sinha 2020; Swain et al. 2022); in one of the studies, Recurrent Neural Networks (RNN) and LSTM algorithms were used together (Srivastava et al. 2023); in one only the LSTM algorithm (Palanimeera and Ponmozhi 2023); one using the 3D‐CNN algorithm (Jain et al. 2021); two using transfer learning algorithms (Garg et al. 2022; Ashraf et al. 2023); one using the Y_PN‐MSSD algorithm (Upadhyay et al. 2023) was found to be used. In one of the studies using ML models, Logistic Regression (LR), Support Vector Machine (SVM), Random Forest (RF), K‐Nearest Neighbours (KNN) and Naïve Bayes (NB) algorithms were used together (Bahukhandi and Gupta 2021); in another, the KNN algorithm was used (Bhosale et al. 2022); in another, EpipolarPose, OpenPose, PoseNet and MediaPipe were used together (Kishore et al. 2022). In one of the studies using both DL and ML models, SVM, KNN, RF, Multi‐Layer Perceptron (MLP), CNN and LSTM algorithms were used together (Anand Thoutam et al. 2022), and in another, MobileNetV2, DenseNet201, SVM and RF algorithms were used together (Rathikarani et al. 2022). In one of them, CNN and SVM algorithms were used together (Gajbhiye et al. 2022) (Table 2).

In all included studies, the statistical method used for yoga pose was accuracy (Yadav et al. 2019; Kumar and Sinha 2020; Bahukhandi and Gupta 2021; Jain et al. 2021; Anand Thoutam et al. 2022; Bhosale et al. 2022; Gajbhiye et al. 2022; Garg et al. 2022; Kishore et al. 2022; Rathikarani et al. 2022; Swain et al. 2022; Ashraf et al. 2023; Palanimeera and Ponmozhi 2023; Srivastava et al. 2023; Upadhyay et al. 2023), while 3 studies also used precision, recall and F1 score (Bahukhandi and Gupta 2021; Garg et al. 2022; Swain et al. 2022) and one study also paid attention to the Area Under the Curve (AUC) curve (Swain et al. 2022) (Table 2).

### 3.4 Methodological Quality Assessment Results

The methodological quality of the included studies was assessed using the JBI Critical Appraisal Checklist for Diagnostic Test Accuracy Studies. Based on this evaluation, 10 out of 15 studies (67%) were classified as high quality (score ≥ 7), 3 studies (20%) as moderate quality (score 5–6) and 2 studies (13%) as low quality (score < 5).

The most common methodological weaknesses identified included: the absence of a pre‐specified accuracy threshold in AI model validation, inadequate reporting of sample selection criteria and insufficient blinding between the index test and the reference standard.

The certainty of evidence was assessed using the GRADE (Grading of Recommendations, Assessment, Development and Evaluations) system. Overall, 6 studies (40%) provided high‐certainty evidence, 5 studies (33%) moderate‐certainty evidence and 4 studies (27%) low‐certainty evidence.

Lower certainty ratings were attributed to high risk of bias, result inconsistencies and small sample sizes. These limitations should be carefully considered when interpreting the findings. Table 3 presents a summary of the GRADE assessment for all included studies.

**TABLE 3:** GRADE evidence.

Study	Risk of bias	Inconsistency	Indirectness	Imprecision	Publication bias	GRADE certainty
Yadav et al. 2019	Low	Low	Low	Low	Low	High
Kumar and Sinha 2020	Low	Low	Low	Moderate	Low	High
Jain et al. 2021	Moderate	Low	Low	Moderate	Moderate	Moderate
Swain et al. 2022	Low	Moderate	Low	Low	Low	High
Garg et al. 2022	Moderate	Moderate	Moderate	Moderate	Moderate	Moderate
Palanimeera and Ponmozhi 2023	High	High	High	High	High	Low
Upadhyay et al. 2023	Low	Low	Low	Low	Low	High
Ashraf et al. 2023	High	High	High	High	High	Low
Srivastava et al. 2023	Moderate	Moderate	Moderate	Moderate	Moderate	Moderate
Bahukhandi and Gupta 2021	Low	Low	Low	Low	Low	High
Bhosale et al. 2022	Moderate	Moderate	Moderate	Moderate	Moderate	Moderate
Kishore et al. 2022	High	High	High	High	High	Low
Anand Thoutam et al. 2022	Moderate	Moderate	Moderate	Moderate	Moderate	Moderate
Rathikarani et al. 2022	Low	Low	Low	Low	Low	High
Gajbhiye et al. 2022	High	High	High	High	High	Low

### 3.5 Results From the Studies

As shown in Table 2, the analysed studies reported real‐time yoga asana prediction accuracy ranging from 92.34% to 99.92% for DL models, 90.9% to 98.51% for ML models and 91.49% to 99.58% for studies employing both methods.

Yadav et al. (2019) achieved 98.92% accuracy in the real‐time prediction of six yoga poses using CNN and LSTM models. Kumar and Sinha (2020) reported 99.92% accuracy with similar models. Jain et al. (2021) conducted a study where they classified 10 yoga poses with 99.39% accuracy using 3D‐CNN. Swain et al. (2022) achieved 99.53% accuracy with CNN and LSTM models while reporting the AUC value as 0.99. Garg et al. (2022) developed the YogaConvo2d model, which outperformed other models with a 99.62% accuracy rate. Palanimeera and Ponmozhi (2023) achieved 99.02% accuracy in real‐time yoga pose prediction using LSTM. Upadhyay et al. (2023) predicted seven yoga poses with 99.88% accuracy using the Y_PN‐MSSD model. Ashraf et al. (2023) achieved 95% accuracy with the YoNet model. Srivastava et al. (2023) reported 92.34% accuracy with RNN and LSTM models. Bahukhandi and Gupta (2021) achieved the best result with 94% accuracy with the logistic regression model. Bhosale et al. (2022) reported 98.51% accuracy using the KNN algorithm. Kishore et al. (2022) reported 90.9% accuracy with MediaPipe. Anand Thoutam et al. (2022) achieved 98.58% accuracy with CNN and 99.38% accuracy with LSTM. Rathikarani et al. (2022) reported 91.49% accuracy with MobileNetV2 and RF models. Gajbhiye et al. (2022) achieved a 98.68% accuracy rate with the CNN model (Table 2).

## 4 Discussion

### 4.1 Main Findings

This systematic review analysed 15 studies on the real‐time prediction of correct yoga asanas in healthy individuals using AI techniques. Various DL and ML algorithms were utilised for real‐time pose estimation, demonstrating high accuracy rates (90.9%–99.92%).

Training AI algorithms necessitates large datasets, cross‐validation and thresholding techniques, which are essential for achieving high accuracy. The reviewed studies indicate that AI‐based yoga pose prediction models successfully learn from structured datasets, demonstrating their potential in automated yoga posture correction and assessment.

In response to the research question, ‘Which AI techniques are used to predict the correct yoga asanas?’, the studies utilised various algorithms, including CNN (Yadav et al. 2019; Kumar and Sinha 2020; Gajbhiye et al. 2022; Swain et al. 2022), LSTM (Yadav et al. 2019; Kumar and Sinha 2020; Swain et al. 2022; Palanimeera and Ponmozhi 2023; Srivastava et al. 2023) and 3D‐CNN (Jain et al. 2021). Additionally, studies employed ML models such as LR (Bahukhandi and Gupta 2021), SVM (Bahukhandi and Gupta 2021; Anand Thoutam et al. 2022; Gajbhiye et al. 2022; Rathikarani et al. 2022), RF (Bahukhandi and Gupta 2021; Anand Thoutam et al. 2022; Rathikarani et al. 2022) and KNN (Bahukhandi and Gupta 2021; Anand Thoutam et al. 2022; Bhosale et al. 2022). Furthermore, MediaPipe, OpenPose, PoseNet and EpipolarPose were used for pose estimation (Kishore et al. 2022).

In response to the second research question, ‘How accurate is the real‐time prediction of correct yoga asanas in healthy individuals using AI techniques?’, DL and ML models demonstrated accuracy rates ranging from 90.9% to 99.92%. These results are consistent with previous studies (Borthakur et al. 2023; Jadhav et al. 2023; Janardhana et al. 2023; Talaat 2023), where similar algorithms demonstrated high classification performance.

### 4.2 Comparison With Existing Literature

Previous research substantiates the effectiveness of AI in yoga pose classification and correction. For instance, Jadhav et al. (2023) introduced LGDeep, a CNN‐based model integrating Xception, VGGNet and SqueezeNet architectures, which outperformed prior approaches. Similarly, Patel and Lathigara (2022) utilised MediaPipe to track whole‐body movements, successfully detecting 33 2D landmarks to estimate yoga poses accurately.

Wang et al. (2019) introduced a Yoga Pose Verification System that analysed users' yoga poses and provided feedback to correct inappropriate postures by detecting 10 key points of the body. The system achieved successful results in classifying four different yoga asanas, with SVM outperforming RF among ML models.

Findings from the present review align with Wu et al. (2021), who highlighted that deep learning's ability to extract hierarchical features from complex body movements enhances AI's capability in pose classification. Furthermore, Gupta and Gupta (2021) emphasised the role of motion sensors in improving AI‐based yoga monitoring systems, supporting the feasibility of automated pose tracking.

KNN consistently demonstrated high accuracy in yoga pose classification among ML models. Bahukhandi and Gupta (2021) reported a 98.51% accuracy using KNN, while Lee et al. (2015) identified four yoga postures in children using KNN and achieved a 93.1% accuracy. Nagalakshmi and Mukherjee (2021) also developed a KNN classifier model with the Euclidean distance function, obtaining a 99.01% accuracy across 13 yoga asanas.

DL models, particularly CNN and transfer learning approaches, exhibited the highest prediction accuracy across multiple studies. Chaudhari et al. (2021) reported 95% classification accuracy using CNN, reinforcing the advantages of DL architectures over traditional ML models.

### 4.3 Implications for Clinical Practice and Research

This systematic review highlights the transformative role of AI‐based yoga pose recognition in clinical practice. AI‐powered posture analysis offers promising applications in rehabilitation, physiotherapy, injury prevention and digital health. In therapy settings, AI can support clinicians by tracking patient progress and ensuring correct movement execution. Real‐time feedback may reduce the risk of re‐injury and improve adherence. These systems can also be integrated into tele‐rehabilitation platforms, enabling remote monitoring and personalised exercise guidance.

From a preventive health perspective, AI‐enabled yoga tools can detect incorrect postures before they lead to injury, which is particularly beneficial for beginners practising at home. When linked with wearable devices and mobile applications, these systems can continuously monitor flexibility, balance and postural stability, offering practical value for older adults, athletes and individuals with movement limitations.

For broader adoption and effectiveness, future research must address current gaps. A key need is to train AI models on more diverse populations to ensure generalisability across body types, ages and levels of mobility. Additionally, standardised evaluation frameworks are essential to allow comparison between models, as current studies vary in terms of performance metrics used, such as accuracy, precision, recall and F1‐score.

There is also a need for longitudinal studies to evaluate whether AI‐based yoga assistants can adapt to users over time, providing individualised feedback that reflects biomechanical changes and progress. Most existing studies are limited to single‐session evaluations, making it unclear whether such systems are sustainable for long‐term use.

Privacy and ethical concerns must also be prioritised. Since many AI models rely on image or video data, issues related to user privacy, biometric data protection and informed consent become critical. Privacy‐preserving approaches, including on‐device processing and encrypted data storage, should be further developed and implemented to build trust and support ethical deployment in healthcare environments.

In summary, integrating AI‐based pose recognition into telemedicine platforms can improve accessibility to remote physiotherapy and rehabilitation. Developing systems that not only classify yoga poses but also offer personalised correction based on biomechanical analysis will significantly enhance the impact of these tools. Designing AI‐supported yoga programmes tailored to specific populations, such as older adults, pregnant women, or individuals with chronic musculoskeletal conditions, will improve safety and effectiveness. Moreover, combining pose estimation with physiological monitoring—such as heart rate variability or breath analysis—may further advance AI's role in mind–body interventions. Addressing these challenges will enable AI‐driven yoga pose recognition systems to become indispensable tools in precision healthcare, digital fitness and preventive medicine.

### 4.4 Strengths and Limitations

This systematic review comprehensively evaluates AI‐based techniques for yoga asana prediction, incorporating both deep learning (DL) and machine learning (ML) approaches. Unlike previous reviews, it includes both real‐time and static pose classification models, offering insights relevant to research and practice. By analysing a wide range of AI architectures, the study deepens understanding of model performance and applicability.

A major strength is the rigorous methodology, including adherence to PRISMA 2020 and the use of JBI and GRADE tools to assess quality and certainty. The comparative analysis of diverse models (e.g., CNN, LSTM, SVM, KNN) provides practical guidance for selecting appropriate AI methods in digital health and yoga‐based interventions.

Nonetheless, several limitations should be noted. The exclusion of recently developed or non‐English studies may limit the inclusion of emerging models and introduce publication bias. The focus on accuracy metrics without evaluating usability or user experience is another gap. Future research should incorporate user‐centred evaluations and real‐world implementation analyses.

The lack of a meta‐analysis due to methodological heterogeneity—variations in AI models, datasets and evaluation metrics—precluded quantitative comparison. Standardised frameworks are needed for consistent evaluation across studies.

Most included studies involved small, homogeneous samples, limiting generalisability. Additionally, real‐world factors such as varying lighting, camera angles and body types were often not considered. Privacy and ethical concerns, particularly regarding video‐based data and biometric security, also require attention. On‐device AI processing may offer a solution.

Finally, long‐term AI model adaptability was not assessed. Most studies focused on single‐session evaluations, leaving questions about sustained performance and user‐specific learning unaddressed. Future work should explore longitudinal validation and model evolution over time.

## 5 Conclusion

This systematic review analysed AI‐based techniques for real‐time prediction of correct yoga asanas in healthy individuals. The DL models employed included CNN, LSTM, 3D‐CNN, Y_PN‐MSSD and RNN, while transfer learning models encompassed CNN‐based InceptionResNetV2, InceptionV3, VGG16, NASNetMobile, YogaConvo2d, ResNet‐50, InceptionNet, Xception and YoNet. Regarding ML models, LR, SVM, RF, KNN, NB and MLP were frequently utilised. Additionally, EpipolarPose, OpenPose, PoseNet and MediaPipe demonstrated high accuracy in predicting yoga postures. Notably, studies utilising only DL models exhibited higher prediction accuracy (ranging from 92.34% to 99.92%) compared to those employing only ML models (90.9% to 98.51%) or a combination of DL and ML techniques (91.49% to 99.58%). In this context, two research questions from the study were answered.

Given the methodological variability among the included studies, careful interpretation of the overall findings is warranted. While most studies demonstrated high methodological quality, the presence of lower‐quality studies highlights potential limitations, such as sample selection bias and inconsistency in reference standards. Future research should prioritise standardised methodologies, the inclusion of larger and more diverse datasets, and the implementation of rigorous validation techniques to improve the reliability of AI‐based yoga asana prediction models.

The GRADE evaluation revealed variability in the overall certainty of evidence across studies. While the majority provided high or moderate certainty, a subset exhibited low certainty due to factors such as high risk of bias, inconsistencies and imprecise study designs. Future research should aim for **more standardised methodologies and larger sample sizes** to improve evidence quality.

AI‐driven real‐time prediction of correct yoga postures constitutes a significant area of research. The integration of AI techniques into yoga practice offers substantial health benefits and holds transformative potential in healthcare. This advancement may enable healthcare professionals, including nurses and physicians, to extend their practice into home‐based interventions. Maintaining correct yoga postures is well documented to enhance health outcomes and reduce disease burden. It is recommended to use DL and ML models for real‐time prediction of yoga asanas in healthy individuals.

The findings underscore the efficacy of AI in enhancing yoga practice by achieving high accuracy in pose prediction. However, variations in methodology, including pose complexity and sample size, impact outcomes. Future research should prioritise the standardisation of methodologies and the integration of multimodal data to enhance consistency and generalisability. Limitations, including biases in AI algorithms and a lack of diverse samples, were identified. These findings highlight the potential of AI in promoting safe and effective yoga practice, particularly in nursing interventions.

## Disclosure

This study is an expanded and revised version of the study “Real‐Time Prediction of Correct Yoga Asanas in Healthy Individuals with Artificial Intelligence Techniques: A Systematic Review” was presented as an oral presentation at The 4th International 7th National Transcultural Nursing Congress held on 30 November‐2 December 2023.

## Ethics Statement

The authors have nothing to report.

## Conflicts of Interest

The authors declare no conflicts of interest.

## Supporting information


Data S1.

## Data Availability

Data sharing not applicable to this article as no datasets were generated or analysed during the current study.
