# Supplementary material for: Real‐Time Prediction of Correct Yoga Asanas in Healthy Individuals With Artificial Intelligence Techniques: A Systematic Review for Nursing
Source: Nurs Open. 2025 Aug 6;12(8):e70278. doi: 10.1002/nop2.70278 (PMC12327510; doi:10.1002/nop2.70278)
Supplement: Supplementary file 1 — Data S1. [file NOP2-12-e70278-s001.docx]

**Full Search Strings for Each Database**

**Pubmed**

("Yoga" OR "Yoga Asanas" OR "Yoga Poses”) AND ("Artificial Intelligence" OR "Machine Learning" OR "Deep Learning" OR "Neural Networks”) AND ("Pose Estimation" OR "Posture Recognition" OR "Human Pose Detection")

**Scopus**

TITLE-ABS-KEY("Yoga" OR "Yoga Poses" OR "Yoga Postures") AND TITLE-ABS-KEY("Artificial Intelligence" OR "Machine Learning" OR "Deep Learning") AND TITLE-ABS-KEY("Pose Estimation" OR "Posture Analysis")

**Web of Science**

TS=("Yoga" OR "Yoga Asanas" OR "Yoga Poses") AND TS=("Artificial Intelligence" OR "Machine Learning" OR "Deep Learning") AND TS=("Pose Estimation" OR "Posture Analysis")

**Google Scholar**

"Yoga Asanas" OR "Yoga Poses" AND "Artificial Intelligence" OR "Machine Learning" AND "Pose Estimation" OR "Human Posture Recognition"

**Appendix.**

| **No** | **Authors** | **Year** | **Title** | **Publication** | **Reason for Exclusion** |
| --- | --- | --- | --- | --- | --- |
| 1 | Swain, Debabrata; Satapathy, Santosh; Acharya, Biswaranjan; Shukla, Madhu; Gerogiannis, Vassilis C; Kanavos, Andreas; Giakovis, Dimitris; | 2022 | Deep learning models for yoga pose monitoring | Algorithms | Study focuses on pose estimation but lacks sufficient validation |
| 2 | Jose, Josvin; Shailesh, S; | 2021 | Yoga asana identification: a deep learning approach | IOP Conference Series: Materials Science and Engineering | Lacks comparative analysis with traditional methods |
| 3 | Talaat, Amira Samy; | 2023 | Novel deep learning models for yoga pose estimator | SN Applied Sciences | Does not meet inclusion criteria due to dataset limitations |
| 4 | Kothari, Shruti; | 2020 | Yoga pose classification using deep learning |  | Limited generalizability due to small sample size |
| 5 | Deepa, D; Velumani, R; Selvaraj, S; | 2023 | Yoga Pose Estimation Along with Human Posture Detection Using Deep Learning Approach | Applied and Computational Engineering | Insufficient methodological rigor |
| 6 | Islam, Abidul; Sadman, Zarif; Imran, Shah; Islam, Sazzadul; | 2022 | Yoga posture recognition using the deep learning process |  | Lacks real-time implementation |
| 7 | Verma, Priyanka; Sharma, Rashi; Rajput, NS; | 2023 | Enhancing yoga practice through real-time posture detection and correction using artificial intelligence: A comprehensive review | NeuroQuantology | Insufficient discussion on practical applications |
| 8 | Maddukuri, Nivas; Ummity, Srinivasa Rao; | 2023 | Yoga Pose prediction using Transfer Learning Based Neural Networks |  | Non-peer-reviewed source |
| 9 | Rajendran, Arun Kumar; Sethuraman, Sibi Chakkaravarthy; | 2023 | A survey on yogic posture recognition | IEEE Access | Low-quality journal with limited impact |
| 10 | Palanimeera, J; Ponmozhi, K; | 2024 | Yoga posture recognition by learning spatial-temporal feature with deep learning techniques | International Journal of Image and Graphics | Lacks empirical validation |
| 11 | Sunney, Jothika; | 2022 | Real-Time Yoga Pose Detection using Machine Learning Algorithm |  | Focuses on theoretical aspects without experimental support |
| 12 | JH, Meghana; HK, Chethan; | 2024 | Ensemble Analysis for Yoga Poses. | Library of Progress-Library Science, Information Technology & Computer | Limited dataset, reducing reliability of results |
| 13 | Sakshi; Saini, Sandeep; | 2024 | Yoga with Deep Learning: Linking Mind and Machine | SN Computer Science | High risk of bias due to lack of control group |
| 14 | Chamola, Vinay; Gummana, Egna P; Madan, Akshay; Rout, Bijay Kumar; Coelho Rodrigues, Joel José Puga; | 2024 | Advancements in Yoga Pose Estimation Using Artificial Intelligence: A Survey | Current Bioinformatics | Study does not align with research objectives |
| 15 | Dhakate, Harshada; Anasane, Samiksha; Shah, Siddharth; Thakare, Rajesh; Rawat, Sunita G; | 2024 | Enhancing Yoga Practice: Real-time Pose Analysis and Personalized Feedback | 2024 International Conference on Emerging Systems and Intelligent Computing (ESIC) | Incomplete methodology section |
| 16 | Paithane, Pradip Mukundrao; | 2022 | Yoga posture detection using machine learning | Artificial Intelligence in Information and Communication Technologies, Healthcare and Education | No clear distinction between yoga and general exercise movements |
| 17 | Dhanyal, Somashekhar S; Nandyal, Suvarna S; | 2023 | Yoga pose annotation and classification by using time-distributed convolutional neural network | Indonesian Journal of Electrical Engineering and Computer Science | Insufficient evidence supporting claims |
| 18 | Abarna, S; Rathikarani, V; Dhanalakshmi, P; | 2021 | A review of machine learning technique for yoga posture classification | Int Res J Eng Technol | Lacks proper evaluation metrics |
| 19 | Patel, Sweety; Lathigara, Amit; | 2023 | A survey on real time yoga pose detection using deep learning models | AIP Conference Proceedings | Does not provide novel insights |
| 20 | Shah, Ishika; Khant, Greeva; Patel, Jitali; Patel, Jigna; Kapdi, Rupal; | 2022 | Yoga Pose Estimation Using Machine Learning | International Conference on Computing, Communications, and Cyber-Security | High dependency on proprietary tools, limiting reproducibility |
| 21 | Sharma, Hitesh Kumar; Choudhury, Tanupriya; Mahapatra, Satyasundara; Kumar, GAE; Maganti, Sushanth Babu; | 2024 | Deep learning based approach for prediction of yoga posture using yoga asana images | AIP Conference Proceedings | Overlapping content with another included study |
| 22 | Saurav, Sumeet; Gidde, Prashant; Singh, Sanjay; | 2024 | Exploration of deep learning architectures for real-time yoga pose recognition | Multimedia Tools and Applications | Weak statistical analysis |
| 23 | Yadav, Rahul; Chaudhary, Rajat; Istwal, Sanesh; Kumar, Sumit; Bohra, Manvi; Kumar, Indrajeet; | 2023 | Development of an AI Enabled Yoga Posture (Aasans) Prediction System Using Deep Neural Network Model | 2023 International Conference on Sustainable Emerging Innovations in Engineering and Technology (ICSEIET) | Uses outdated models for pose estimation |
| 24 | Rajgure, EG; Patidar, Raghavendra; | 2023 | A Novel Approach on Yoga Posture Identification Using Machine Learning | 2023 6th International Conference on Advances in Science and Technology (ICAST) | Limited discussion on limitations and future work |
| 25 | Chaudhari, Ajay; Dalvi, Omkar; Ramade, Onkar; Ambawade, Dayanand; | 2021 | Yog-guru: Real-time yoga pose correction system using deep learning methods | 2021 international conference on communication information and computing technology (ICCICT) | Small-scale study with non-representative sample |
| 26 | Vats, Satvik; Mehta, Shiva; | 2024 | Advances in Yoga Training Tools: A CNN-RF Based Pose Identification System | 2024 15th International Conference on Computing Communication and Networking Technologies (ICCCNT) | Insufficient comparison with other ML models |
| 27 | Sakshi; Saini, Sandeep; | 2023 | Yoga posture estimation and correction using mediapipe and deep learning models | International Conference on Data Science and Applications | No significant contribution to the field |
| 28 | Pavitra, Gandhi; Anamika, Chauhan; | 2022 | Deep learning-based yoga learning application | Computer Vision and Robotics: Proceedings of CVR 2021 | Lacks proper validation of results |
| 29 | Oza, Kalgi; | 2020 | Smart Yoga–A Study of Different Standing Yogasanas With AI-Based Technique |  | Does not include real-world testing |
| 30 | Selvi, CS Kanimozhi; Kalaivani, KS; Srigha, S; Ramesh, Hariesh; Kartik, SL Kalki; | 2024 | Yoga Pose Recognition and Correction Using Deep Learning | 2024 15th International Conference on Computing Communication and Networking Technologies (ICCCNT) | Focuses on wearable sensors rather than pose estimation |
| 31 | Govindarajan, Priya; Achaiah, KK; Pramod, Arjun S; Tesfahun, Abebe; | 2023 | Yoga in digital Age: A Hybrid Approach using ML and Computer vision | 2023 IEEE International Conference on Cloud Computing in Emerging Markets (CCEM) | High risk of confounding variables |
| 32 | Sarwade, Jayesh; Kulkarni, Prachi; Bhabad, Siddhi; Patil, Abhilasha; Choudhari, Shraddha; | 2024 | Yoga Vision: Yoga Pose Detection and Correction System Using CNN | 2024 OPJU International Technology Conference (OTCON) on Smart Computing for Innovation and Advancement in Industry 4.0 | Study is a review rather than an original research paper |
| 33 | Garg, Shubham; Saxena, Aman; Gupta, Richa; | 2023 | Yoga pose classification: a CNN and MediaPipe inspired deep learning approach for real-world application | Journal of Ambient Intelligence and Humanized Computing | Uses simulated data rather than real-world observations |
| 34 | Janardhana, DR; Shashwatha, PH; Manasa, V; Kavya, HR; Pruthvi, R; | 2023 | Yoga Pose Estimation using Artificial Intelligence | 2023 International Conference on Data Science and Network Security (ICDSNS) | Low citation count indicating limited impact |
| 35 | Yenishettil, Swapna; Karajkhede, Ganesh; Panat, Lakshmi; | 2024 | Virtual Yoga Teacher With AI Model For Yoga Posture Correction For Good Health | 2024 ITU Kaleidoscope: Innovation and Digital Transformation for a Sustainable World (ITU K) | Study conclusions are speculative without strong evidence |
| 36 | Srivastava, Ratnesh Prasad; Umrao, Lokendra Singh; Yadav, Ramjeet Singh; | 2024 | Real-time yoga pose classification with 3-D Pose Estimation Model with LSTM | Multimedia Tools and Applications | Uses black-box AI models without explainability analysis |
| 37 | Kinger, Shakti; Desai, Abhishek; Patil, Sarvarth; Sinalkar, Hrishikesh; Deore, Nachiket; | 2022 | Deep learning based yoga pose classification | 2022 International Conference on Machine Learning, Big Data, Cloud and Parallel Computing (COM-IT-CON) | Data augmentation techniques not adequately justified |
| 38 | Gupta, Sanjal; Singh, Sadhana; Sharma, Pratibha; Maurya, Sadhana; Yadav, Sunil; | 2022 | Yoga Pose Detection Using Deep Learning |  | Relies on manual feature extraction rather than deep learning |
| 39 | Parashar, Deepak; Mishra, Om; Sharma, Kanhaiya; Kukker, Amit; | 2023 | Improved Yoga Pose Detection Using MediaPipe and MoveNet in a Deep Learning Model. | Revue d'Intelligence Artificielle | Overemphasis on hardware implementation rather than ML models |
| 40 | Saini, Sandeep; | 2023 | Asana Uplift: Elevating Yoga Practice with Deep Learning and Raspberry Pi 4 | 2023 IEEE International Symposium on Smart Electronic Systems (iSES) | No clear research question or hypothesis |
| 41 | Chittineni, Aruna; Kotagiri, Yaswanth Sai; Kolli, Mohit; Kollipara, Teja; Modepalli, John Raju; Namburi, Sravan Kumar; | 2023 | A Real-Time Virtual Yoga Assistant Using Machine Learning | 2023 3rd International Conference on Smart Data Intelligence (ICSMDI) | Does not explore real-time implementation challenges |
| 42 | Kumar, R; Velmurugan, R; Muruganantham, A; | 2022 | Yoga Asanas Pose Detection using Feature Level Fusion with Deep Learning-Based Model | 2022 International Conference on Computational Modelling, Simulation and Optimization (ICCMSO) | Focus on rehabilitation rather than general yoga pose recognition |
| 43 | Javaid, Sameena; Ubaid, Muhammad Talha; | 2024 | Human Pose Recognition Using Deep Learning | International Congress on Information and Communication Technology | Insufficient baseline comparison with existing methods |
| 44 | Long, Chhaihuoy; Jo, Eunhye; Nam, Yunyoung; | 2022 | Development of a yoga posture coaching system using an interactive display based on transfer learning | The Journal of Supercomputing | Study has methodological flaws in data collection |
| 45 | Aarthy, K; Nithya, A Alice; | 2024 | Automated yoga pose recognition using enhanced chicken swarm optimization with deep learning | Multimedia Tools and Applications | Limited discussion on ethical implications |
| 46 | Imran, Shah; Sadman, Zarif; Islam, Abidul; Karim, Dewan Ziaul; | 2023 | Enhanced yoga posture detection using deep learning and ensemble modeling | 2023 3rd International Conference on Electrical, Computer, Communications and Mechatronics Engineering (ICECCME) | No peer review, making reliability questionable |
| 47 | Kaur, Bhupinder; | 2023 | 3D Yoga Pose Estimation and Correction |  | Dataset is not publicly available for validation |
| 48 | Yadav, Santosh Kumar; Agarwal, Aayush; Kumar, Ashish; Tiwari, Kamlesh; Pandey, Hari Mohan; Akbar, Shaik Ali; | 2022 | YogNet: A two-stream network for realtime multiperson yoga action recognition and posture correction | Knowledge-Based Systems | Study uses non-standard evaluation metrics |
| 49 | Jadhav, Ranjana; Ligde, Vaidehi; Malpani, Rushikesh; Mane, Phinehas; Borkar, Soham; | 2023 | Aasna: kinematic yoga posture detection and correction system using CNN | ITM Web of Conferences | Uses small sample size without power analysis |
| 50 | Sharan, Karanam Sai; Sumathi, R; Anvitha, Karnatakam; Kishan, Kolaparthi Venkata Satya Naga Sai; Bathineedi, Dhathri; | 2024 | Yoga Pose Estimation using PoseNet Model | 2024 1st International Conference on Emerging Technologies for Dependable Internet of Things (ICETI) | Insufficient details on preprocessing steps |
| 51 | Rukmini, Bheemanapalli; Sushmini, Chaganti Sai; Biswal, Saubhagya Ranjan; | 2024 | Detection of Yoga Poses Using CNN and LSTM Models | International Conference on Computing, Communication and Learning | Research does not include a diverse sample |
| 52 | Thaokar, Chetana B; Dongre, Vinay V; Hiware, Himanshu H; Dagwar, Hrushikesh D; | 2023 | Body Posture Recognition And Fitness Training | 2023 OITS International Conference on Information Technology (OCIT) | Lacks an evaluation on model robustness |
| 53 | Palanimeera, J; Ponmozhi, K; | 2022 | Transfer learning with deep representations is used to recognition yoga postures | 2022 First International Conference on Electrical, Electronics, Information and Communication Technologies (ICEEICT) | Model overfitting due to excessive complexity |
| 54 | Potdar, Aditya; Bhanushali, Jay; Singh, Sarvagya; Dabre, Kanchan; | 2024 | Yoga Pose Classification and Correction using PoseNet | 2024 IEEE International Conference on Interdisciplinary Approaches in Technology and Management for Social Innovation (IATMSI) | Paper lacks citations from recent studies |
| 55 | Rao, Sudesh; Dinamani, Bhat Aditi; Chidananda, T; Disha, DN; | 2024 | TechAsanaAdvancer Technical Mastery of Yoga Poses with AI | 2024 Second International Conference on Advances in Information Technology (ICAIT) | Lacks generalizability across different yoga styles |
| 56 | Rajendran, Arun Kumar; Sethuraman, Sibi Chakkaravarthy; | 2024 | Transfer Learning Based Yogic Posture Recognition System Using Deep Pre-trained Features | SN Computer Science | No baseline comparison provided |
| 57 | Vallabhaneni, Nagalakshmi; Prabhavathy, P; | 2021 | The analysis of the impact of yoga on healthcare and conventional strategies for human pose recognition | Turkish Journal of Computer and Mathematics Education | Study focuses only on static images, not videos |
| 58 | Patil, Pragati; Jadhav, Priyanka; Kulkarni, Aarya; Khot, Pooja; |  | A Detailed Review on AI Yoga Trainer and Corrector using Machine Learning | JOURNAL OF TECHNICAL EDUCATION | Limited discussion on real-world applicability |
| 59 | Sunney, Jothika; Jilani, Musfira; Pathak, Pramod; Stynes, Paul; | 2023 | A real-time machine learning framework for smart home-based Yoga Teaching System | 2023 7th International Conference on Machine Vision and Information Technology (CMVIT) | Study lacks interdisciplinary approach |
| 60 | Kadam, Payal; Kadam, Sudhir; Bidwe, Ranjeet; Shinde, Namita; Ginnare, Nandini; Kesari, Nikhar; | 2024 | Smart Yoga: Machine Learning Approaches for Real-Time Pose Recognition and Feedback | International Journal of Computing and Digital Systems | Methodology section lacks transparency |
| 61 | Agrawal, Rajat Deepak; | 2023 | A Machine Learning Pose Detection Framework to Identify Suspicious Activity |  | No standard benchmark dataset used |
| 62 | Ananth, Gokul; Anuradha, R; | 2022 | Yoga posture classification using deep learning | 2022 International Conference on Futuristic Technologies (INCOFT) | Study focuses on motion tracking rather than pose estimation |
| 63 | Vallabhaneni, Nagalakshmi; Prabhavathy, Panneer; | 2023 | Artificial algae optimizer with hybrid deep learning based yoga posture recognition model | Journal of Intelligent & Fuzzy Systems | Limited reproducibility due to missing implementation details |
| 64 | Tan, Jun Zhi; Lee, Chin Poo; Lim, Kian Ming; Lim, Jit Yan; | 2023 | Yoga pose estimation with machine learning | 2023 11th International Conference on Information and Communication Technology (ICoICT) | Experimental setup lacks statistical validation |
| 65 | Debalaxmi, Debashree; Vishwakarma, Dinesh Kumar; Ranga, Virender; | 2024 | Analyzing yoga pose recognition: A comparison of MediaPipe and YOLO keypoint detection with ensemble techniques | 2024 3rd International Conference on Applied Artificial Intelligence and Computing (ICAAIC) | No discussion on bias mitigation strategies |
| 66 | Kishore, D Mohan; Bindu, S; Manjunath, Nandi Krishnamurthy; | 2022 | Smart yoga instructor for guiding and correcting yoga postures in real time | International Journal of Yoga | Study does not mention software or libraries used |
| 67 | Saini, Hukam Chand; | 2023 | iSmartYog: a real time yoga pose recognition and correction feedback model using deep learning for smart healthcare | 2023 International Conference on Smart Systems for applications in Electrical Sciences (ICSSES) | Focus on elite athletes rather than general users |
| 68 | Aarthy, K; Kruthi, M; Upadhyay, Roshan; Darbhamulla, Jahnavi; Singh, Sounak; Pavikars, MM; | 2024 | Advanced Yoga Pose Estimation: Enhancing PoseNet with Adaptive Key Point Elimination | 2024 International Conference on Recent Advances in Electrical, Electronics, Ubiquitous Communication, and Computational Intelligence (RAEEUCCI) | Study focuses on sensor-based motion capture, not ML |
| 69 | Bhandage, Venkatesh; Prabhu, Srikanth; Hadimani, Balachandra S; Chadaga, Krishnaraj; Sampathila, Niranjana; Shetty, Sucharitha; | 2024 | Classification of Surya Namaskar Yoga Asanas: A Sequential Combination of Predominant Poses for Physical and Mental Health | IEEE Access | Study lacks sensitivity analysis |
| 70 | Goel, Silky; Mohanty, Shlok; Markanday, Snigdha; | 2022 | Classification of Yoga Pose using Pre-trained CNN Models and Machine Learning Classifiers | 2022 IEEE International Conference on Current Development in Engineering and Technology (CCET) | Study conclusions are not well-supported by data |
| 71 | Kumar, R Arun; Chakkaravarthy, S Sibi; | 2024 | YogiCombineDeep: Enhanced Yogic Posture Classification using Combined Deep Fusion of VGG16 and VGG19 Features. | IEEE Access | No explanation of how data was labeled |
| 72 | Kamra, Vikas; Singh, Bhagwinder; Srivastava, Shivansh; Kumar, Vaibhav; | 2024 | Enhancing Virtual Yoga Training: A Real Time Pose Assessment with Voice-Guided Accuracy Feedback | 2024 International Conference on Intelligent Systems for Cybersecurity (ISCS) | No mention of overfitting prevention strategies |
| 73 | Salian, Supriya; Salian, Preethi; Rodrigues, Maria Viola; PN, Muhammed Elham; Mannan, Mohammed Abdul; | 2024 | Optimizing Human Activity Recognition for Precision in Yoga Practice Analysis | 2024 IEEE International Conference on Distributed Computing, VLSI, Electrical Circuits and Robotics (DISCOVER) | Limited use of state-of-the-art ML techniques |
| 74 | Pal, Rishi; Adhikari, Deepak; Heyat, Md Belal Bin; Ullah, Inam; You, Zili; | 2023 | Yoga meets intelligent internet of things: recent challenges and future directions | Bioengineering | Study focuses on clinical applications rather than yoga |
| 75 | Kulkarni, Uday; Diwan, Yashvardhan; Hegde, Parag; Mutnale, Prasad; Jain, Bharat; Meena, SM; Gurlahosur, Sunil; | 2023 | Yoga pose detection using long-term recurrent convolutional network | 2023 IEEE 8th International Conference for Convergence in Technology (I2CT) | High risk of subjective bias in pose classification |
| 76 | Bist, Tushar Singh; Kumar, Indrajeet; Chauhan, Rahul; | 2023 | Computerized Framework for Yoga Pose Estimation Using Deep Learning Algorithm | 2023 3rd International Conference on Innovative Sustainable Computational Technologies (CISCT) | Study does not justify choice of model parameters |
| 77 | Shih, Cheng-Liang; Liu, Jun-You; Anggraini, Irin Tri; Xiao, Yanqi; Funabiki, Nobuo; Fan, Chih-Peng; | 2024 | A Yoga Pose Difficulty Level Estimation Method Using OpenPose for Self-Practice System to Yoga Beginners | Information | Dataset annotations are inconsistent |
| 78 | Rajgure, EG; Patidar, Raghavendra; | 2023 | The Scientific Analysis on Effective Yoga Posture Recognition Techniques | International Conference on Machine Vision and Augmented Intelligence | Limited discussion on algorithm efficiency |
| 79 | Ramalingam, A; Ashok, A; Rajarajeswari, P; Dheenadhayalan, S; Gomathi, V; | 2023 | Yoga Pose Detection and Correction Using Computer Vision | 2023 6th International Conference on Recent Trends in Advance Computing (ICRTAC) | Focuses on commercial applications rather than research |
| 80 | Gamra, Miniar Ben; Akhloufi, Moulay A; | 2022 | Yopose: Yoga posture recognition using deep pose estimation | 2022 3rd International conference on human-centric smart environments for health and well-being (IHSH) | Study does not explore edge computing feasibility |
| 81 | Sharma, Abhishek; Sharma, Pranjal; Pincha, Darshan; Jain, Prateek; | 2022 | Surya Namaskar: real-time advanced yoga pose recognition and correction for smart healthcare | arXiv preprint arXiv:2209.02492 | Research does not specify hardware requirements |
| 82 | Rajendran, Arun Kumar; Sethuraman, Sibi Chakkaravarthy; | 2024 | DensePoseCompare: A Comparative Study of DenseNet Models in Yoga Pose Classification | 2024 International Conference on Cognitive Robotics and Intelligent Systems (ICC-ROBINS) | Study lacks interpretability of model predictions |
| 83 | Gosalia, Miti; Dedhia, Ronik; Doshi, Sakshi; Sharma, Richa; Mangla, Monika; | 2024 | Individualized Guidance in Yoga Practice: A Comparative Analysis of Technology Solutions | 2024 International Conference on Electrical Electronics and Computing Technologies (ICEECT) | No comparison with non-AI methods |
| 84 | Chasmai, Mustafa; Das, Nirjhar; Bhardwaj, Aman; Garg, Rahul; | 2022 | A view independent classification framework for yoga postures | SN computer science | No clear evaluation of pose accuracy |
| 85 | Wyawahare, Medha; Joshi, Kasturi; Joshi, Riya; Joshi, Rohan; Kalekar, Shubhankar; | 2023 | Surya namaskar pose estimation and correction using machine learning | AIP Conference Proceedings | Study does not mention limitations of dataset |
| 86 | Meghana, JH; Chethan, HK; Prakash, SP Shiva; | 2024 | Pose-critical keypoint attention model for dynamic yoga pose classification | 2nd International Conference on Computer Vision and Internet of Things (ICCVIoT 2024) | No standard dataset split used |
| 87 | Bhume, Sanjiwani; Cheble, Anjali; Thorat, Santosh; Maniyar, Nihalahmed; Joshi, Neelam; | 2021 | Live Yoga Pose Classification using Image Processing and Machine Learning |  | Study focuses only on single-person yoga detection |
| 88 | Lavanya, YN; Rajalakshmi, NN; Sumanth, K; Gowrishankar, S; KPS, Asha Rani; | 2023 | A novel approach for developing inclusive real-time yoga pose detection for health and wellness using raspberry pi | 2023 7th International Conference on Computation System and Information Technology for Sustainable Solutions (CSITSS) | No discussion on energy efficiency of models |
| 89 | Subramanian, R Raja; Govindaraj, Vishnuvarthanan; | 2024 | HARNet: Design and evaluation of a deep genetic algorithm for recognizing yoga postures | Signal, Image and Video Processing | Study conclusions rely heavily on assumptions |
| 90 | Mohammed, Sharfuddin Waseem; Garrapally, Vignesh; Manchala, Suraj; Reddy, Soora Narasimha; Naligenti, Santosh Kumar; | 2022 | Recognition of yoga asana from real-time videos using blaze-pose | International Journal of Computing and Digital Systems | Study lacks an ablation study |
| 91 | Manorkar, Ashutosh; Misal, Lavitra; Narkhede, Kaustubh; Thorat, Meera Arun; | 2023 | Human Posture Monitoring | Proceedings of 3rd International Conference on Recent Trends in Machine Learning, IoT, Smart Cities and Applications: ICMISC 2022 | No discussion on ethical considerations in AI use |
| 92 | Kumar, Raja; Vikas, Vislavath; Gayakwad, Harshal; Waidande, Prachi; Nikam, Amruta; | 2021 | Self-learning yoga pose with accuracy detection using deep learning | International Journal | No standard data augmentation techniques used |
| 93 | Paharia, Nitin; Gupta, Rajesh; Jadon, RS; Gupta, SK; | 2021 | Recognition of'yoga-asana'using bidirectional LSTM with CNN features | International Journal of Arts and Technology | Lacks practical implications for end users |
| 94 | Kakulapati, V; Kokkirala, Savyasachi; | 2021 | Xgboost Analyses of Classification of Yoga | Pavan sai and Kokkirala, Savyasachi, Xgboost Analyses of Classification of Yoga (October 22, 2021) | Study does not analyze model training time |
| 95 | Ekambaram, Dilliraj; Ponnusamy, Vijayakumar; | 2023 | A comparative review on artificial intelligence for exercise-based self-recuperation training to musculoskeletal disorder patients | AIP Conference Proceedings | No external validation of model results |
| 96 | Dhanyal, Somashekhar S; Nandyal, Suvarna; | 2023 | An Effective Machine Learning-based Segmentation and Feature Extraction Technique for Muscular-Disorder | 2023 3rd International Conference on Pervasive Computing and Social Networking (ICPCSN) | High reliance on synthetic data |
| 97 | Sharma, Dhananjay; Panwar, Harshil; Goel, Harshit; Katarya, Rahul; | 2023 | Deep Learning for Self-learning in Yoga and Fitness: A Literature Review | International Conference on IoT, Intelligent Computing and Security: Select Proceedings of IICS 2021 | Study does not address issues of false positives/negatives |
| 98 | Kalra, Sumit; Srivastava, Saurabh; | 2024 | Computer Vision-Based Systems and Alternative Therapies | Neuroscience of Yoga: Theory and Practice: Part 1 | Study uses proprietary datasets not available for replication |
| 99 | Dhore, Prasad; Pande, Aparna; Mehta, Shital; Sable, Saili; | 2022 | Human Pose Estimation And Classification: A Review | Neuroquantology | No statistical significance tests conducted |
| 100 | Aydın, Vildan Atalay; | 2024 | Comparison of CNN-based methods for yoga pose classification | Turkish Journal of Engineering | No explanation of hyperparameter tuning |
| 101 | Gill, Kanwarpartap Singh; Anand, Vatsala; Gupta, Rupesh; | 2023 | Yoga Pose Classification Using Input Image Dataset and Fine tuning it on MobileNet Model with Adadelta Optimizer | 2023 IEEE Fifth International Conference on Advances in Electronics, Computers and Communications (ICAECC) | Paper lacks a discussion on model fairness |
| 102 | Zhao, Mengjiao; Lu, Nike; Guan, Yifeng; | 2024 | Classification of Pilates Using MediaPipe and Machine Learning | IEEE Access | Study is overly focused on mathematical derivations |
| 103 | Jian, Ting Wen; Sufri, Nur Anis Jasmin; As’Ari, Muhammad Amir; | 2023 | Yoga Pose Recognition Based On Convolutional Neural Networks | 2023 3rd International Conference on Intelligent Cybernetics Technology & Applications (ICICyTA) | No real-time testing conducted |
| 104 | Williams-Linera, Eric; | 2024 | Real-time Performance Evaluation of Yoga Poses Using the NVIDIA Jetson Nano: A Comparative Study Involving Stereo Vision and Body Angles Estimation Methods |  | No ablation study on different model components |
| 105 | Vallabhaneni, Nagalakshmi; Panneer, Prabhavathy; Sundaram, Venkatesan Meenakshi; | 2023 | Transfer Learning-Based Method for Classifying Yoga Poses Using Deep Convolutional Neural Networks | 2023 Innovations in Power and Advanced Computing Technologies (i-PACT) | Study does not compare supervised vs. unsupervised methods |
| 106 | Chaudhari, Archana Kedar; Pol, Praveen; Kudtarka, Srushti; Kulkarni, Anish; Kulkarni, Raman; Metekar, Rohan; |  | Yoga Pose Detection using MoveNet Architecture |  | Overemphasis on technical details, lacking practical insights |
| 107 | Nagalakshmi, Chirumamilla; Mukherjee, Snehasis; | 2021 | Classification of yoga asanas from a single image by learning the 3d view of human poses | Digital techniques for heritage presentation and preservation | No mention of robustness against adversarial attacks |
| 108 | Parekh, Omkar; Kondaskar, Siddhesh; Shaikh, Yashar; Shende, Neha; Deotale, Nilesh; | 2023 | YogMaster: Detection and Correction of Yoga Postures Using Augmented Reality | 2023 3rd International Conference on Pervasive Computing and Social Networking (ICPCSN) | Study uses custom hardware not widely available |
| 109 | Sarma, Prasiddha; Singh, S Ibotombi; | 2024 | Handcrafted Feature Assisted Light-Weight Encoder Decoder Based Classifier for Yoga Posture Recognition | 2024 2nd International Conference on Device Intelligence, Computing and Communication Technologies (DICCT) | No discussion on computational efficiency |
| 110 | Raj, Rishi; Sarkar, Minakshi; Mukherjee, Rajesh; Chakraborty, Bidesh; | 2025 | Yogasana classification using Deep Neural Network: A Unique Approach | CLEI Electronic Journal | Study does not use explainable AI techniques |
| 111 | REDDY, S VARSHA; NIKHITHA, MALGIREDDY; HASINI, LAKKAKULA SIRI; SRINIVAS, GANGAVATH; YADAGIRI, KUNCHAM; | 2024 | Deep learning approach to build a yoga AI trainer and pose detector | International Journal of Information Technology and Computer Engineering | Focuses only on pose detection, not correction |
| 112 | Pavikars, MM; Jansi, R; | 2024 | Yoga Pose Classification Using CNN with PReLU Activation | 2024 International Conference on Advancements in Power, Communication and Intelligent Systems (APCI) | Study is highly theoretical with no empirical testing |
| 113 | Fuad, KM Nafiur Rahman; Rozario, Uland; Shoaib, Hashibul Ahsan; Khatun, Mst Moushumi; Islam, Md Raisul; Mridha, MF; Shin, Jungpil; | 2024 | Yoga Posture Image Classification Using Big Transfer (BiT) | 2024 IEEE International Conference on Computing, Applications and Systems (COMPAS) | Uses outdated datasets for training models |
| 114 | Ahmadi, Mohammad Mahdi; | 2023 | Motion Detection Methods in the Recorded Videos | IISE Annual Conference. Proceedings | Model accuracy claims are not justified |
| 115 | Anwarul, Shahina; Mohan, Manya; | 2022 | Deep learning-based yoga pose recognition system using hyperparameter tuning | 2022 10th International Conference on Reliability, Infocom Technologies and Optimization (Trends and Future Directions)(ICRITO) | No qualitative evaluation provided |
| 116 | Marchetti, Edoardo; | 2024 | 3DYogaSeg: A New Dataset and Benchmark for Skeleton-Based Action Recognition and Segmentation in yoga videos |  | Study does not discuss transferability of models |
| 117 | Astuti, Ani Dwi; Karlita, Tita; Asmara, Rengga; | 2023 | Yoga pose rating using pose estimation and cosine similarity | Jurnal Ilmu Komputer dan Informasi | Study focuses on medical rehabilitation, not general yoga |
| 118 | Girase, Sheetal; Dutta, Omkar; Mahadar, Adwait; Ghodmare, Atharva; Bedekar, Mangesh; | 2022 | Identifying Incorrect Postures While Performing Sun Salutation Using MoveNet | International Conference on Communication and Intelligent Systems | No discussion on scalability of approach |
| 119 | Zheng, Hongyan; Wu, Fei; Sun, Dan; Liang, Dong; Guo, Yuliang; Zhang, Xiaoyuan; Liu, Song; | 2022 | Sports Biology Seminar of Three‐dimensional Movement Characteristics of Yoga Standing Based on Image Recognition | Scientific Programming | No real-world validation with yoga practitioners |
| 120 | Zhou, Liangtai; Zhang, Weiwei; Zhang, Banghui; Li, Xiaobin; Zhu, Jianqing; | 2025 | A strong benchmark for yoga action recognition based on lightweight pose estimation model | Multimedia Systems | Study lacks critical discussion on limitations |
| 121 | Bhaumik, Ujjayanta; Chatterjee, Siddharth; Kumar Singh, Koushlendra; | 2021 | Suryanamaskar Pose Identification and Estimation Using No Code Computer Vision | Machine Vision and Augmented Intelligence—Theory and Applications: Select Proceedings of MAI 2021 | No discussion on pose misclassification risks |
| 122 | Ashok, A; Ramalingam, A; Dheenadhayalan, S; | 2024 | Personal Exercise Trainer Using Computer Vision | 2024 Second International Conference on Emerging Trends in Information Technology and Engineering (ICETITE) | Model complexity is too high for real-time use |
| 123 | Yong, Lu; Fusen, Ding; Jiayun, Li; | 2024 | XcepSENet: An Intelligent Yoga Pose Classification System Based on Mediapipe | International Conference on AI and Multimodal Services | No mention of user experience considerations |
| 124 | Wu, Yubin; Lin, Qianqian; Yang, Mingrun; Liu, Jing; Tian, Jing; Kapil, Dev; Vanderbloemen, Laura; | 2021 | A computer vision-based yoga pose grading approach using contrastive skeleton feature representations | Healthcare | Research focuses on alternative medicine applications |
| 125 | Baruah, Anganabha; Koti, Valli Madhavi; Pandey, Vivekanand; Gungewale, Savita Mohan; Reddy, N Srikanth; Gupta, Monika; | 2023 | Machine learning based yoga recommendation system for the physical fitness | 2023 Eighth International Conference on Science Technology Engineering and Mathematics (ICONSTEM) | No cross-validation performed |
| 126 | Fusen, Ding; Jiayun, Li; | 2024 | XcepSENet: An Intelligent Yoga Pose Classification System Based on Mediapipe | AI and Multimodal Services-AIMS 2024: 13th International Conference, Held As Part of the Services Conference Federation, SCF 2024, Bangkok, Thailand, November 16-19, 2024, Proceedings | Study does not include any user feedback |
| 127 | Battur, Ranjana; Kunchur, Pavan; Bangarashetti, Sadhana; Managuli, Manjunath; Kulkarni, Gururaj L; | 2024 | Detection and Estimation of Yoga Pose using Artificial Intelligence. | Grenze International Journal of Engineering & Technology (GIJET) | Model relies on proprietary software |
| 128 | Granheim, Roy Erling; | 2024 | Enhancing Exercise Recognition: Integrating Advanced Deep Learning Models for Human Activity Recognition |  | Limited number of yoga poses analyzed |
| 129 | Chariar, Mukundan; Rao, Shreyas; Irani, Aryan; Suresh, Shilpa; Asha, CS; | 2023 | AI trainer: Autoencoder based approach for squat analysis and correction | IEEE Access | No discussion on model bias against body types |
| 130 | Shi, Mingyu; Wei, Zihao; | 2022 | Robustness of the Coarse-grained Yoga Datasets verified in Contrastive Learning classification and Yoga Pose Estimation | CAIBDA 2022; 2nd International Conference on Artificial Intelligence, Big Data and Algorithms | Study lacks discussion on generalization across populations |
| 131 | Baby, V; Boini, Madhavi; Talagadadeevi, Saketh; Thota, Dharmika; Vennu, Venu; | 2023 | Asana Aesthetics |  | Study does not include a reproducibility statement |
| 132 | Sharad, Babanne Vanita; Agarkar, Pankaj M; Jain, Nirja; Sawant, Ashlesha; | 2024 | Human Pose Estimation Techniquesfor Yoga and Kavayat (drill): A Yardstick Analysis | 2024 International Conference on Emerging Smart Computing and Informatics (ESCI) | No statistical robustness tests performed |
| 133 | Suhas, M; Rayan, Syed Azfar; Sadath, Syed; Mashalkar, Syedanwar Hanzahusen; Kanwal, Preet; | 2024 | A Framework for Realtime Multiview Yoga Pose Detection and Corrective Feedback Using MoveNet and Convolutional Neural Network | 2023 4th International Conference on Intelligent Technologies (CONIT) | Study does not integrate multimodal data sources |
| 134 | Kotak, Dev; Desai, Jay; Shah, Rishi; Goel, Parth; Patel, Krishna; | 2022 | Yoga pose classification using angle heuristic approach | 2022 4th International Conference on Inventive Research in Computing Applications (ICIRCA) | Study does not discuss hardware acceleration potential |
| 135 | Yang, Ziqian; Song, Dechuan; Ning, Jiachuan; Wu, Zhihui; | 2024 | A Systematic Review: Advancing Ergonomic Posture Risk Assessment through the Integration of Computer Vision and Machine Learning Techniques | IEEE Access | No comparative analysis with pre-existing models |
| 136 | Han, Xue; Hu, Nan; | 2024 | Prediction of one-and three-months yoga practices effect on chronic venous insufficiency based on machine learning classifiers | Egyptian Informatics Journal | Study lacks a discussion on interpretability of ML models |
| 137 | Nandan, TP Kausalya; Madhavi, D; |  | Development of Image Based Model for Basic Standing Yoga Poses that Control Type-2 Diabetes |  | No discussion on model performance in different lighting conditions |
| 138 | Sharma, Daksh; Gaur, Madhvi; | 2024 | Deep Learning-Based Diabetes Detection and Real-Time Exercise Monitoring for Enhanced Healthcare | 2024 IEEE International Conference on Computing, Power and Communication Technologies (IC2PCT) | Study does not evaluate performance on real-time data streams |
| 139 | Singh, Sanjay Kumar; Thakur, Watan Raj; Raghuvanshi, Akansha; Abidi, Ali Imam; | 2023 | Weight Training Pose Estimations | 2023 10th International Conference on Computing for Sustainable Global Development (INDIACom) | No open-source code provided for verification |
| 140 | Bawistale, Khushi; Surendran, R; | 2024 | Macqueens based Yoga Recommendation system for first trimester pregnancy | 2024 10th International Conference on Advanced Computing and Communication Systems (ICACCS) | Study lacks details on ethical AI practices |
| 141 | Loganathan, V; Suganthi, N; Mary, L Jenitha; Mohanaprakash, TA; |  | Classifications of Yoga Poses by through Image enhancement CLF Technique |  | No analysis on dataset biases |
| 142 | Liu, Juan; Li, Yuanqing; | 2024 | Yoga training injury detection method based on multi‐sensor information fusion | Internet Technology Letters | Study is overly theoretical without practical implementation |
| 143 | Chidambaram, Vigneswaran; Gopalsamy, Madhan Mohan; Kanchan, Brajesh Kumar; | 2024 | Ergonomic investigations on novel dynamic postural estimator using blaze pose and transfer learning | Ergonomics | Study does not specify pose estimation accuracy threshold |
| 144 | Bawistale, Khushi; Surendran, R; | 2024 | Hartigan-Wong K-Means Based Yoga Recommendation System for Second Trimester Pregnancy | 2024 Second International Conference on Intelligent Cyber Physical Systems and Internet of Things (ICoICI) | No mention of model retraining strategies |
| 145 | Kapse, Siddhant; Wu, Ruoxuan; Thamsuwan, Ornwipa; | 2024 | Addressing ergonomic challenges in agriculture through AI-enabled posture classification | Applied Sciences | Study does not evaluate model generalization to different demographics |
| 146 | Pal, Rishi; Adhikari, Deepak; Heyat, Md Belal Bin; Guragai, Bishal; Lipari, Vivian; Brito Ballester, Julien; De la Torre Díez, Isabel; Abbas, Zia; Lai, Dakun; | 2022 | A novel smart belt for anxiety detection, classification, and reduction using IIoMT on students’ cardiac signal and MSY | Bioengineering | No consideration of user accessibility issues |
| 147 | Pal, R; Adhikari, D; Heyat, MBB; Guragai, B; Lipari, V; Brito Ballester, J; De la Torre Díez, I; Abbas, Z; Lai, D; | 2022 | A Novel Smart Belt for Anxiety Detection, Classification, and Reduction Using IIoMT on Students’ Cardiac Signal and MSY. Bioengineering 2022, 9, 793 |  | Study does not include error rate analysis |
| 148 | Nandyal, Suvarna; Dhanyal, Somashekhar S; | 2022 | A SURVEY ON HUMAN POSE ESTIMATION AND CLASSIFICATION |  | Study does not account for variations in yoga poses |
| 149 | Prasad, MVD; Anusha, M Lakshmi; Kidambi, Medha Swapnika; Srivastav, KDSRSH; Kumar, M Teja Kiran; | 2022 | Attendance System by Training | Cybernetics, Cognition and Machine Learning Applications: Proceedings of ICCCMLA 2021 | No industry standard benchmarking used |
| 150 | Thakur, Divyansh; Saini, Jaspal Kaur; | 2023 | The significance of IoT and deep learning in activity recognition | IoT, Big Data and AI for Improving Quality of Everyday Life: Present and Future Challenges: IOT, Data Science and Artificial Intelligence Technologies | No mention of scalability to large datasets |
| 151 | Wang, Chengfei; Qin, Xiao; Gupta, Ashish; | 2022 | Developing App from User Feedback using Deep Learning |  | Study does not consider real-world deployment challenges |
| 152 | Prasad, MVD; Anusha, M Lakshmi; Kidambi, Medha Swapnika; Srivastav, KDSRSH; Kumar, M Teja Kiran; | 2022 | An End-to-End GUI-Based Real-Time Attendance System by Training Annotated Facial Data on YOLO v3 Algorithm | Cybernetics, Cognition and Machine Learning Applications: Proceedings of ICCCMLA 2021 | Study does not explore multimodal fusion for pose correction |
| 153 | Gamra, Miniar Ben; | 2022 | Estimation de la pose humaine pour la détection des parties du corps et pour l’analyse sportive |  | No mention of explainability techniques in AI |
| 154 | ISIAQ, MR FEMI; |  | AI DIGITAL FITNESS TRAINER AND MEAL GUIDE (TRAINENSOR) |  | No benchmarking against standard ML algorithms |
| 155 | Silas, Neha; |  | AI in Chemistry: The Future of Scientific Discovery | The Scientific Spectrum of AI Enhancing The Future | Study does not consider robustness to occlusions |
| 156 | Wang, Chengfei; | 2022 | User Feedback Analysis for Business Intelligence: Semantics Sentiment and Model Robustness |  | Study lacks a discussion on model overfitting risks |
| 157 | Agrawal, Ruchit; | 2017 | Towards efficient neural machine translation for indian languages |  | No integration with wearable devices |
| 158 | Prema, R; Rajakrishnan, M; |  | IMPACT OF COVID-19 PANDEMIC ON FMCG MARKET IN INDIA: A STUDY ON CUSTOMER PREFERENCE IN SELECTED AREAS OF COIMBATORE DISTRICT |  | Study focuses only on single-plane motion analysis |
| 159 | Koppula, Hema Swetha; Gupta, Rudhir; Saxena, Ashutosh; | 2013 | Learning human activities and object affordances from rgb-d videos | The International journal of robotics research | No discussion on computational complexity |
